# Supplementary material for: Iceberg melting substantially modifies oceanic heat flux towards a major Greenlandic tidewater glacier
Source: Nat Commun. 2020 Nov 25;11:5983. doi: 10.1038/s41467-020-19805-7 (PMC7689500; doi:10.1038/s41467-020-19805-7)
Supplement: Supplementary file 1 — Supplementary Information [file 41467_2020_19805_MOESM1_ESM.pdf]

Supplementary information for: 'Iceberg melting substantially modifies oceanic heat flux towards a major Greenlandic tidewater glacier'

Introduction

This auxiliary material supports the results, discussion and conclusions presented in the main text. Supplementary Text 1 provides further detail on the methodology and reasoning underlying the generation of icebergs in our model, and is supported by Supplementary Fig. 1. Supplementary Figs. 2-10 and Supplementary Tables 1-3 are not discussed here but support the arguments in the main text. Figures are included in the order they are mentioned in the main text.

Supplementary Text 1: Iceberg distribution

For our model, it is necessary to prescribe both the location of icebergs within the model domain, and their dimensions. To achieve this, the user provides the exponent of the power law describing the size-frequency distribution of icebergs throughout the fjord as whole, the maximum draught (or length) of the icebergs to be generated (i.e. the upper limit in the size-frequency distribution), and a 2-D array of desired iceberg cover (at the fjord surface) in plan-view — that is, the proportion of each cell in plan-view that is to be taken up by icebergs. This array is used to calculate the total area of the fjord surface that should be taken up by icebergs (henceforth the fjord-wide target area). Each of these inputs are available in the form of existing observations[1] or can be idealised. We then generate the fjord-wide size-frequency distribution such that the total surface area of the generated icebergs in plan-view is within 1% of the fjord-wide target area. Individual icebergs from this distribution are allocated to cells throughout the fjord, in such a way as to minimise the difference with the desired iceberg cover (Supplementary Fig. 1). Throughout this process, we account for the bathymetry of the fjord (i.e. icebergs cannot extend below the seafloor), which (in the setup used here) generally resulted in the deeper icebergs being placed close to the head of the fjord (Supplementary Fig. 1).

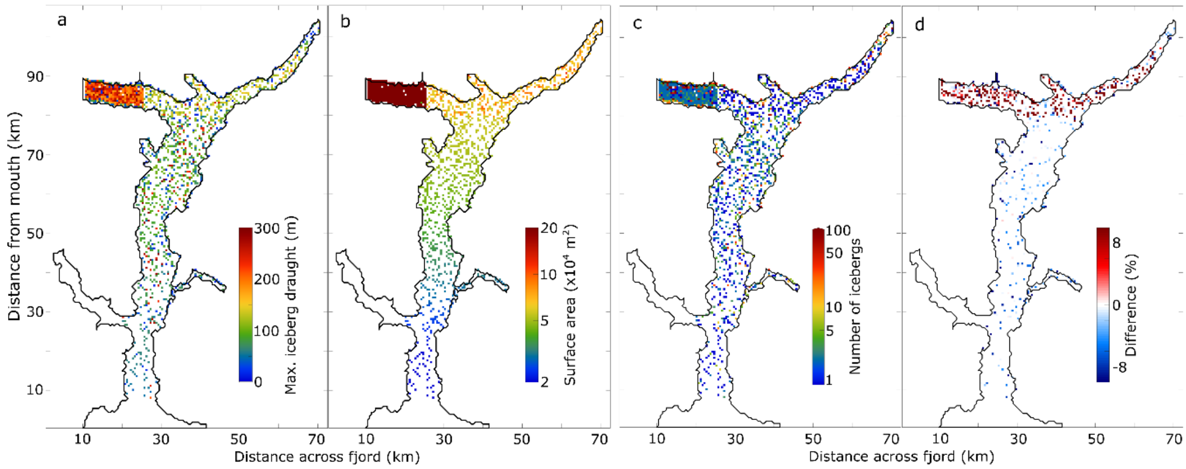

Supplementary Figure 1. Iceberg distributions in Sermilik Fjord. (a) Maximum iceberg draught in each cell. (b) Derived iceberg surface area at the fjord surface in plan-view. (c) Number of icebergs per cell. (d) Difference between derived and target iceberg surface area, as a percentage of the target surface area, with positive values indicating that the derived surface area was greater than the target.

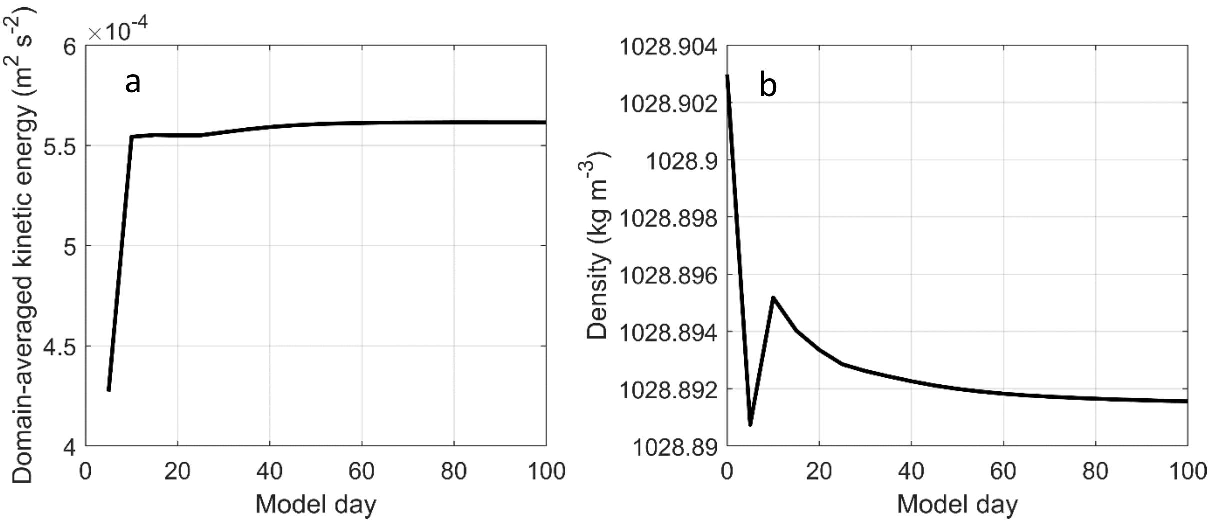

Supplementary Figure 2. Example assessment of simulation steady-state conditions from the summer runoff forcing scenario. (a) Domain-averaged kinetic energy. (b) Depth-averaged modelled density through a water column in the middle of the fjord.

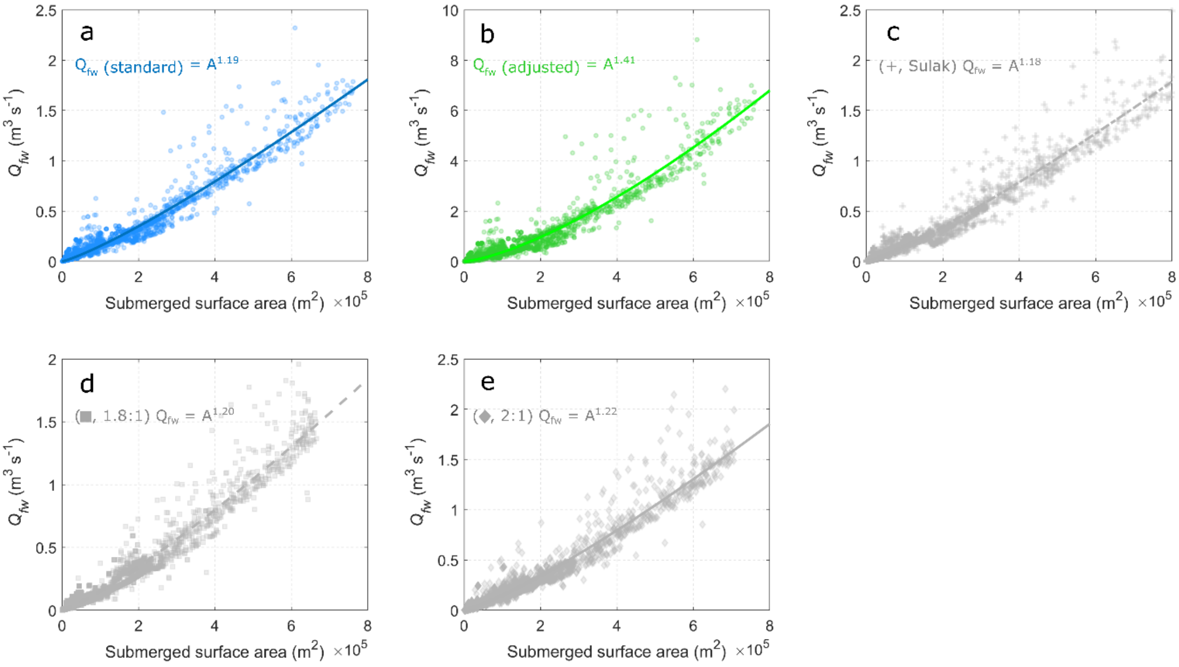

Supplementary Figure 3. Relationship between submerged iceberg surface area and freshwater flux for individual icebergs. All plots show simulations with 'channelised' subglacial hydrology and 1200 m^3 s^{-1} runoff with: (a) standard melt rate parameter values (i.e. the summer runoff forcing scenario), (b) adjusted melt rate parameter values (Jackson et al.[2]), (c) volume-area relationship of Sulak et al.[1], (d) length to keel depth ratio of 1.8:1 and, (e) length to keel depth ratio of 2:1. Note the differing y-axis scales between plots.

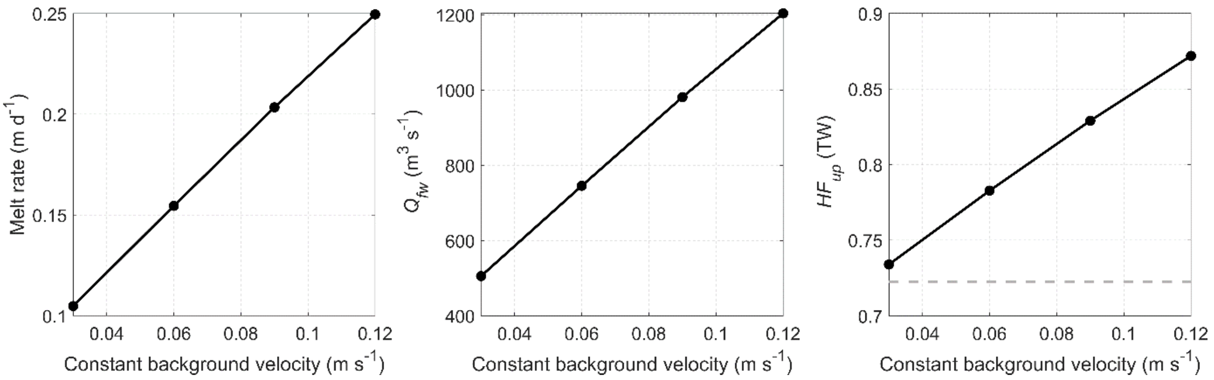

Supplementary Figure 4. Sensitivity of key results to constant background velocity at iceberg-ocean interface. The dashed grey line in the right panel is the up-fjord heat flux across the mélange flux gate in the no-iceberg scenario (location in Fig. 1a of the main text).

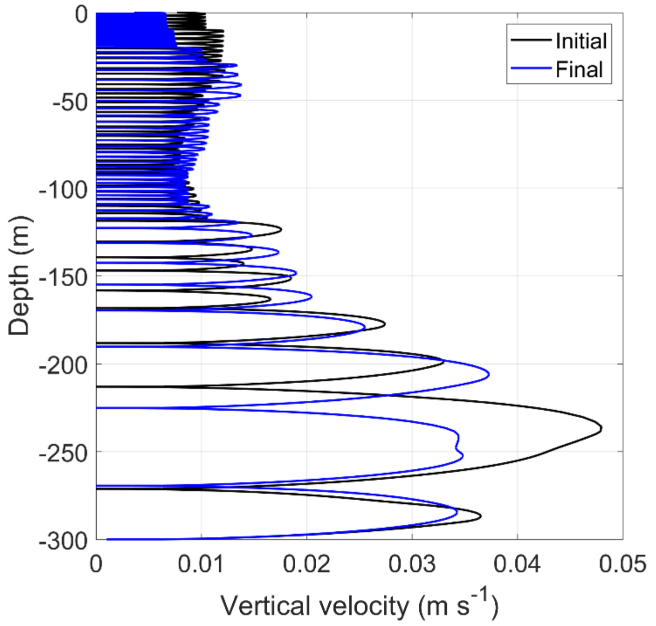

Supplementary Figure 5. Depth-varying background velocity used to bound sensitivity simulations. Background velocities were estimated using the line plume model of Jenkins (2011), which was bounded by the initial conditions (black line) and domain-averaged final conditions (blue line) of the summer runoff forcing scenario.

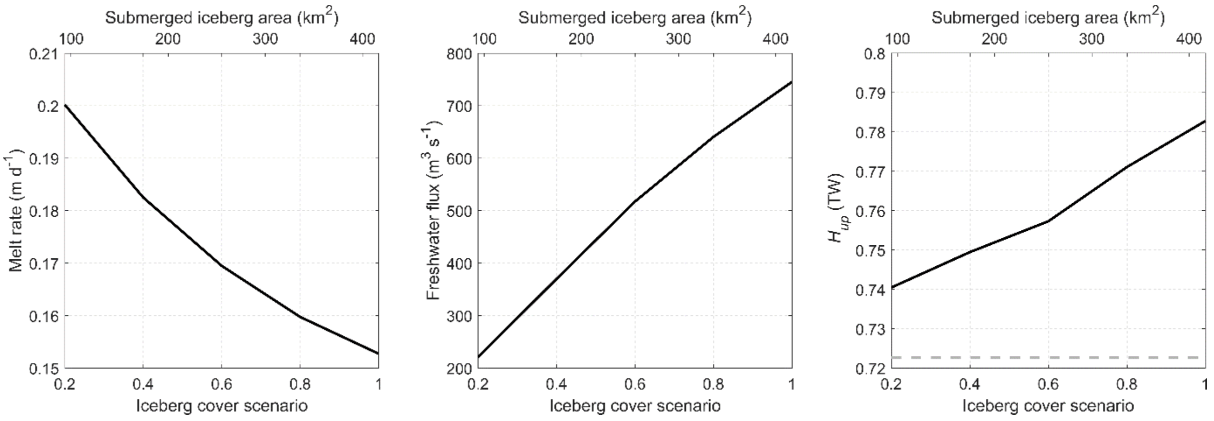

Supplementary Figure 6. Sensitivity of key results to maximum iceberg cover and submerged surface area. Iceberg cover scenarios 0.2-0.8 have approximately 20-80% of the plan-view iceberg surface area as the primary simulations discussed in them main text (cover scenario 1 here). The dashed grey line in the right panel is the up-fjord heat flux across the mélange flux gate in the no-iceberg scenario (location in Fig. 1a of the main text).

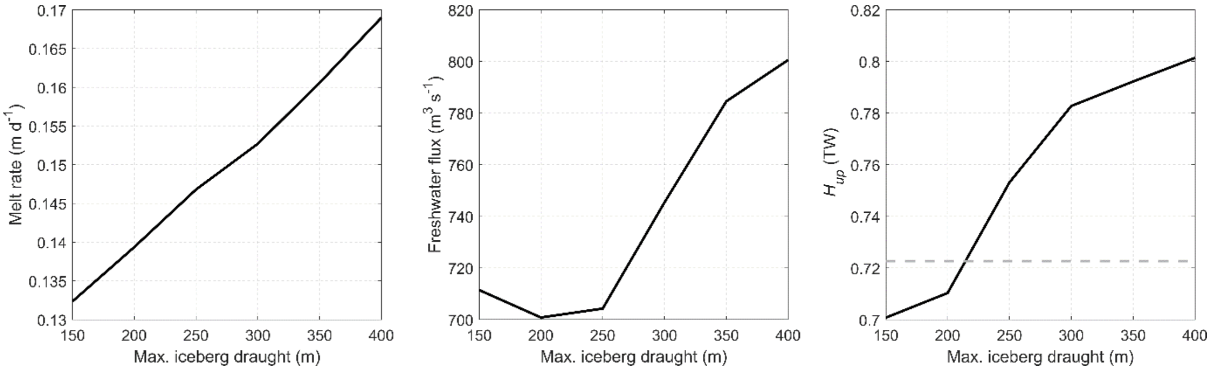

Supplementary Figure 7. Sensitivity of key results to maximum iceberg draught. The dashed grey line in the right panel is the up-fjord heat flux across the mélange flux gate in the no-iceberg scenario (location in Fig. 1a of the main text).

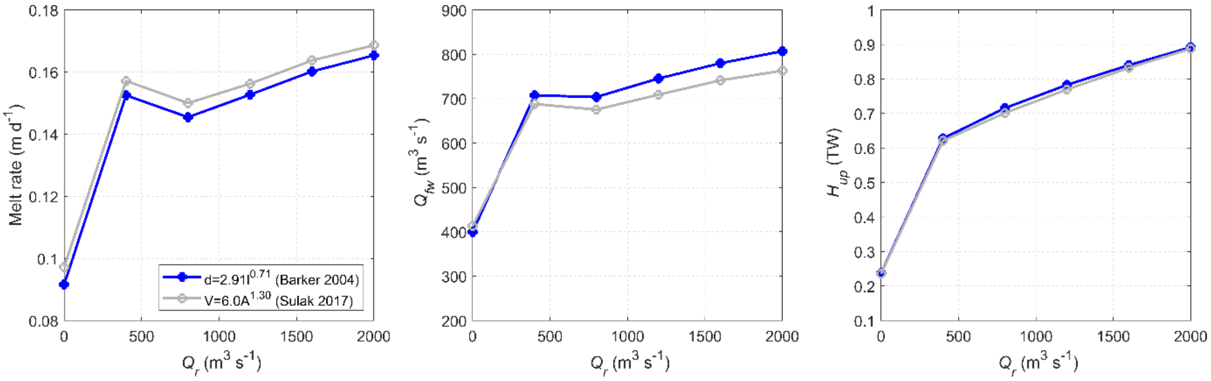

Supplementary Figure 8. Sensitivity to iceberg aspect ratio used. The aspect ratio of Barker[3] was used in the primary simulations discussed in the main text.

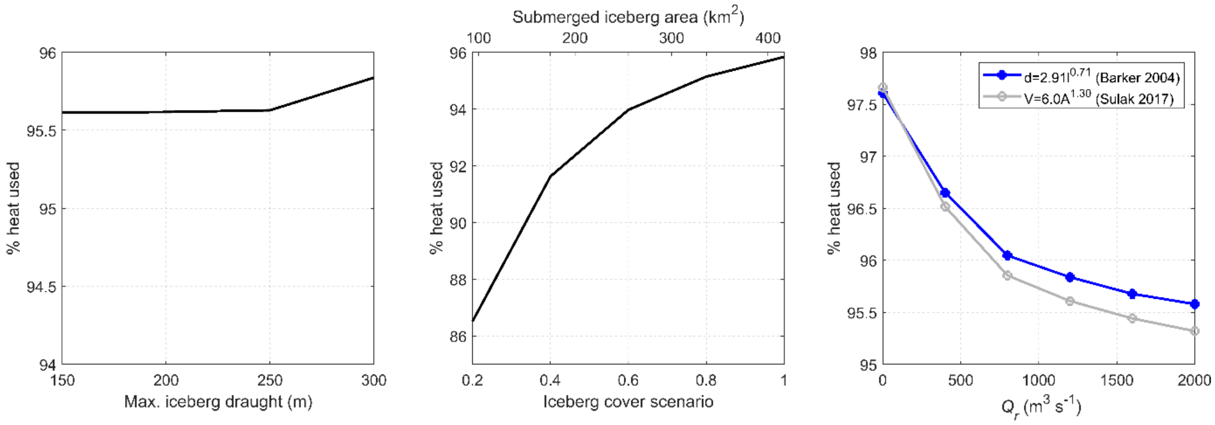

Supplementary Figure 9. Sensitivity of heat used by icebergs to iceberg geometry. As in Fig. 6 of the main text, the y-axis values are the proportion of the heat used for ice melt in each of the domains that is used by icebergs. Iceberg cover scenarios 0.2-0.8 have approximately 20-80% of the plan-view iceberg surface area as the primary simulations discussed in them main text (cover scenario 1 here). Note the different y-axis scales on each of the panels.

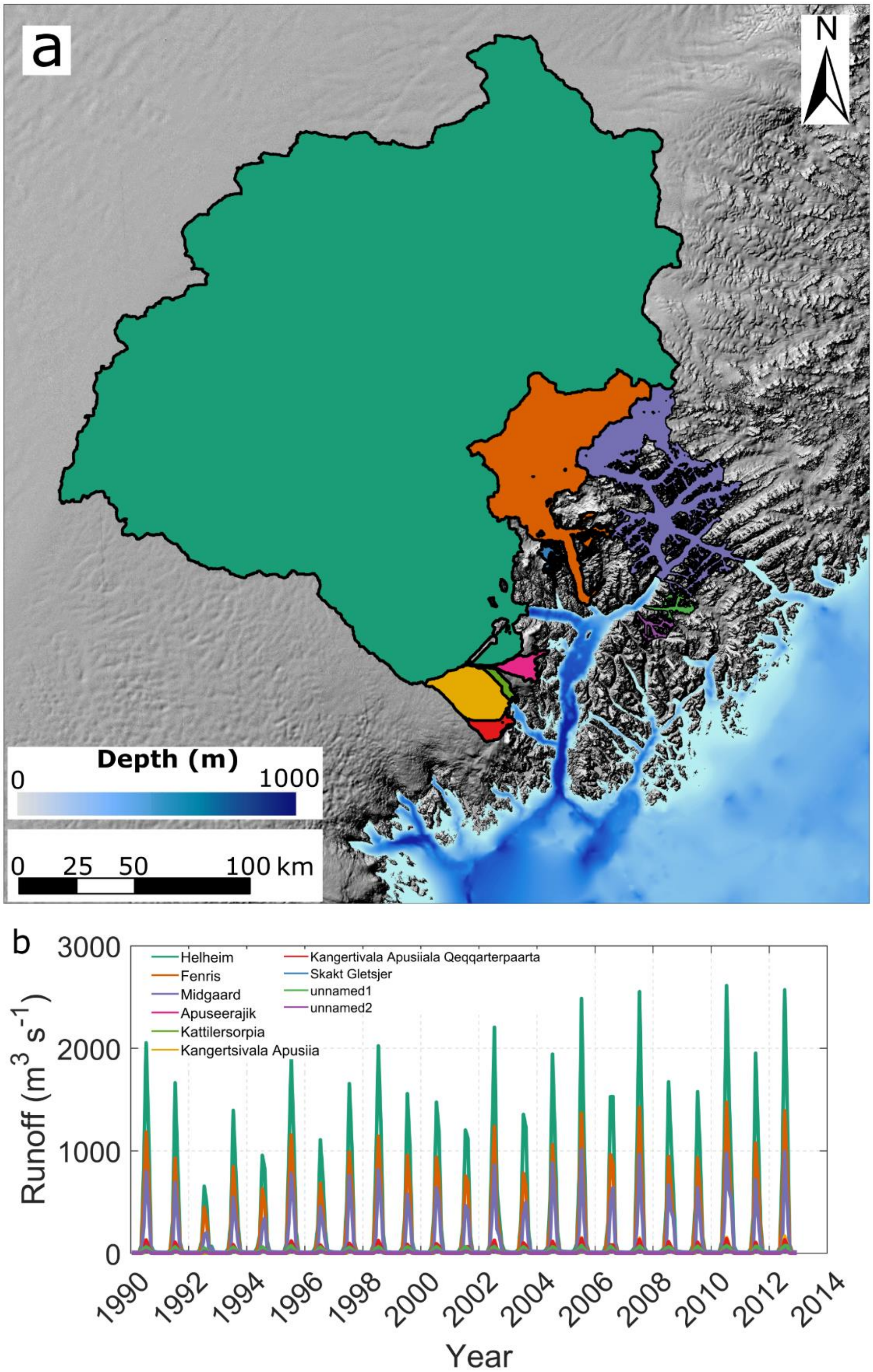

Supplementary Figure 10. Glacier drainage basins and runoff in Sermilik Fjord. (a) Subglacial hydrological basins (coloured shapes) overlaid on the Greenland Ice Mapping Project Digital Elevation Model[4] and BedMachine v3[5] bathymetry (blue shading). (b) Estimated subglacial discharge at indicated glacier termini during 1990-2012, based on RACMO2.3 runoff[6] and simple hydrological routing (Methods). Glacier names in (b) are from Bjørk et al.[7]. The colours in (b) correspond to the drainage basin colours in (a).

Symbol	Description	Value *(adjusted)*	Units
C_i	Heat capacity of ice	2000	J kg^{-1} °C^{-1}
C_w	Heat capacity of water	3974	J kg^{-1} °C^{-1}
α	Entrainment parameter	0.10	
g	Acceleration due to gravity	9.81	m s^{-2}
T_i	Ice temperature	-10	°C
L	Latent heat of melting	3.34 x 10^{5}	J kg^{-1}
Γ_T	Thermal turbulent transfer coefficient	2.20 x 10^{-2} *(4.40 x 10^{-2})*	
Γ_S	Salt turbulent transfer coefficient	6.20 x 10^{-4} *(1.24 x 10^{-3})*	
C_d	Drag coefficient	0.0025 *(0.01)*	
λ_1	Freezing point slope	-5.73 x 10^{-2}	°C psu^{-1}
λ_2	Freezing point offset	8.32 x 10^{-2}	°C
λ_3	Freezing point depth	-7.61 x 10^{-4}	°C m^{-1}
ρ_o	Reference density	1027	kg m^{-3}
U_{bg}	Background velocity	0.06	m s^{-1}
f	Coriolis parameter	1.37 x 10^{-4}	rad s^{-1}
ρ_i	Ice density	917	kg m^{-3}
$diffK_zT,S$	Vertical Laplacian diffusion coefficient for temperature and salinity	1 x 10^{-5}	m^2 s^{-1}
$diffK_hT,S$	Horizontal Laplacian diffusion coefficient for temperature and salinity	30	m^2 s^{-1}
$viscA_z$	Vertical eddy viscosity	1 x 10^{-5}	m^2 s^{-1}
viscC2Smag	Smagorinksy non-dimensional viscosity factor	2.2	

Supplementary Table 1. Model parameters. Note the thermal and salt turbulent transfer coefficients and the drag coefficient have ‘adjusted’ values in brackets.

	Total freshwater flux (m^3 s^{-1})	Contribution of melting below pycnocline to total freshwater flux (%)	Up-fjord heat flux across mélange flux gate (TW)
**Summer runoff forcing**	745	37.0	0.78
Depth-varying background velocity			
**Initial**	399	28.4	0.72
**Final**	386	26.4	0.72
Size-frequency distribution			
**-1.8**	767	41.4	0.78
**-1.9**	765	39.8	0.79

Supplementary Table 2. Summary of key results to a depth-varying background velocity, and iceberg size-frequency distribution. Background velocities are based on line plume simulations bounded by the summer runoff forcing scenario 'initial' and 'final' conditions (Supplementary Fig. 5).

Suite		Submerged iceberg area (km^2)
no-icebergs		0
full observed cover	Depth=2.91*length$^{0.71}$ (Barker 2004 - standard)	416.77
	Depth=2.91*length$^{0.71}$ (Barker 2004 - adjusted)	416.77
	Volume = 6.0*Area$^{1.30}$ (Sulak 2017)**	390.35
	Length : keel depth ratio = 2:1*	486.57
	Length : keel depth ratio = 1.8:1*	528.36
	SFD -1.8*	423.90
	SFD -1.9*	431.01
	Max draught 150 m*	412.23
	Max draught 200 m*	398.31
	Max draught 250 m*	401.65
	Max draught 350 m*	422.09
	Max draught 400 m*	413.94
	Sensitivity: background velocity*	416.77
$c_{0.2}$*		93.74
$c_{0.4}$*		174.87
$c_{0.6}$*		266.70
$c_{0.8}$*		350.89

Supplementary Table 3. Summary of simulations and associated submerged iceberg surface area. SFD simulations used different exponents on the power laws used to generate iceberg size-frequency distributions. Simulations $c_{0.2\text{-}0.8}$ had approximately 20-80% of the iceberg cover as the primary simulations (Barker 2004). Simulations with a * were only run using 1200 m^3 s^{-1} runoff and 'channelised' drainage (Methods). Simulation suites with a ** were run using all runoff values and 'channelised' drainage.

References

1. Sulak, D. J., Sutherland, D. A., Enderlin, E. M., Stearns, L. A. & Hamilton, G. S. Iceberg properties and distributions in three Greenlandic fjords using satellite imagery. *Ann. Glaciol.* 1–15 (2017). doi:10.1017/aog.2017.5

2. Jackson, R. H. *et al.* Meltwater Intrusions Reveal Mechanisms for Rapid Submarine Melt at a Tidewater Glacier. *Geophys. Res. Lett.* **47**, (2020).

3. Barker, A., Sayed, M. & Carrieres, T. Determination of iceberg draft, mass and cross-sectional areas. *Proc. 14th Int. Offshore Polar Eng. Conf.* 899–904 (2004).

4. Howat, I. M., Negrete, A., and Smith, B. E. The Greenland Ice Mapping Project (GIMP) land classification and surface elevation data sets. *Cryosphere* **8**, 1509-1518 (2014).

5. Morlighem, M. *et al.* BedMachine v3: Complete bed topography and ocean bathymetry mapping of Greenland from multi-beam echo sounding combined with mass conservation. *Geophys. Res. Lett.* **44**, 11051–11061 (2017).

6. Noël, B. *et al.* Modelling the climate and surface mass balance of polar ice sheets using RACMO2 - Part 1: Greenland (1958-2016). *Cryosphere* **12**, 811–831 (2018).

7. Bjørk, A. A., Kruse, L. M., and Michaelsen, P. B. Brief Communication: Getting Greenland's glaciers right – a new data set of all official Greenlandic glacier names. *Cryosphere* **9**, 2215-2218 (2015).
